# A Public health wound: health and work among children engaged in the worst forms of child labour in the informal sector in Dhaka, Bangladesh: a retrospective analysis of Médecins Sans Frontières occupational health data from 2014 to 2023

**DOI:** 10.1186/s12889-025-22483-z

**Published:** 2025-04-15

**Authors:** Grazia Caleo, Sohana Sadique, Didem Yuce, Martins Dada, Bianca Benvenuti, Jobin Joseph, Debbie Malden, Kalyan Velivela, Salim Mahmud Chowdhury, Christabel Mayienga, Tasmia Shenjuti, Gayathrie Sadacharamani, Rezwanur Rahman Masum, Mark Sherlock, Hamza Atim, Patrick Keating

**Affiliations:** 1 Médecins Sans Frontières, London, UK; 2 Médecins Sans Frontières, Dhaka, Bangladesh; 3 Médecins Sans Frontières, Amsterdam, The Netherlands; 4 Médecins Sans Frontières, Nairobi, Kenya; 5 Centre for Injury Prevention and Research, Dhaka, Bangladesh

**Keywords:** Child labour, Occupational health, Work-related injuries, Bangladesh

## Abstract

**Background:**

Bangladesh has the second highest burden of child labour in South Asia. The informal sector employs most of the children however, data on health including injuries and place of work for children are limited. As the deadline for the Sustainable Development Goals to end child labour is upon us, it is paramount to document the impact of child labour on health. This study aims to contribute to this knowledge gap by presenting medical data from occupational health clinics (OHCs) set up by Médecins Sans Frontières (MSF) in a peri-urban area of Dhaka, Bangladesh.

**Methods:**

We did a retrospective analysis of health care records of children attending MSF OHCs between February 2014 and December 2023. We stratified the analysis by sex and age (< 14 years and ≥ 14- < 18 years). We looked at morbidities according to type of factory, whether children reported working with machinery, and examined nutritional and mental health (2018–2023) status.

**Results:**

Over the study period, there were 10,200 occupational health consultations among children < 18 years, of which 4945 were new/first time consultations. The average age of children attending their first consultation was 14.7 years, of which 61% were male. Fifteen percent reported living inside the factory. Children worked in all prohibited categories of the informal sector. Almost all children reported operating machinery. Musculoskeletal (26%) and dermatological (20%) were the most identified conditions, and 7.5% of consultations were for work-related injuries. A higher proportion of male children had injuries (11% vs 2.5% in girls). Children working in metal factories accounted for most injuries (65%). Mood-related disorders accounted for 86% of the 51 mental health consultations. Half of all children were malnourished with higher levels in boys and those < 14 years.

**Conclusions:**

Findings suggest that children face hazardous realities; engaged in the worst form of labour, bearing important morbidity and injury burden, with vulnerabilities varying by sex and age. Despite their economic contributions to the informal sector, they remain largely invisible and exploited. This study highlights the urgent need for child rights-based research and cross-sectoral approaches that actively involve children to develop sustainable, targeted solutions to eliminate child labour.

**Supplementary Information:**

The online version contains supplementary material available at 10.1186/s12889-025-22483-z.

## Introduction

*“ There are many factories where child workers have to do heavy work like the welding factory. They have to weld the iron during day or night. They have to work using hammer or sometimes in front of the fire. This scenario is extreme for those child workers.*

*As we (factory owners) we treat them (children/adolescents) a bit rudely and as he is a child so it hurts him, otherwise if he would be adult it would not impact that much. ….But there are many (factory) owners who torture the child workers.*

*Female workers face some harassment. You understood what type of harassment I meant. Some of them do not mention about the harassment thinking of they might lose their job so, it’s a fear for them’’. (Metal factory owner,* Kamrangirchar a peri-urban area in Dhaka, Bangladesh*)–* [1].

This is one of the common narratives Médecins Sans Frontières (MSF) teams heard about children working in Kamrangirchar, a peri-urban area in Dhaka, Bangladesh. Narratives corroborated by observational experience and occupational health medical services provided over the last 10 years by MSF in this area, where an unknown number of children under 18 years old are employed in the informal sector and involved in recycling, metal, plastic, leather and garment sectors [2].

Child labour is a violation of children’s rights, and despite the ambition to end it by 2025, the number of children working is increasing worldwide from 151·6 million in 2016 to 160 million in 2020 [3]. Suboptimal global statistics report that another 22,000 children are deemed to be killed at work every year [4].

From a public health perspective, child labour and its consequences hinder the achievement of Sustainable Development Goals (SDG) 8 (Decent Work and Growth) and 16.2 ( Protect Children from Abuse, Exploitation, Trafficking and Violence) and is considered a harmful practice. By inflicting physical, emotional and mental health ‘’wounds’’ on children, it is associated with negative health outcomes in the long and short term, creating social vulnerabilities that are perpetuated in vicious cycles over generations (Table 1) [5–8].

**Table 1:** Key concepts on child labour [9, 10]

**Child labour**: Child labour is any work that deprives children of their childhood, their potential and their dignity. It interferes with children’s education and negatively affects their emotional, developmental and physical well-being. **Worst forms of child labour (WFCL):** The WFCL are a subset of child labour, defined by the ILO Convention No. 182 as **prohibited for all children under the age of 18 years and are to be eliminated as a matter of urgency**. They include: • all forms of slavery or practices similar to slavery, such as the sale and trafficking of children, debt bondage and serfdom and forced or compulsory labour, including forced or compulsory recruitment of children for use in armed conflict; • the use, procuring or offering of a child for prostitution, for the production of pornography or for pornographic performances; • the use, procuring or offering of a child for illicit activities, in particular for the production and trafficking of drugs as defined in relevant international treaties; • work which, by its nature or the circumstances in which it is carried out, is likely to harm the health, development, safety or morals of a child (also called: “**hazardous work”**). **Hazardous Work:** is work which by its nature or the circumstances in which it is carried out, is likely to harm the health, development, safety or morals of children. **It is one of the worst forms of child labour and is therefore prohibited for all children under the age of 18 years**. ILO Recommendation No. 190 urges governments to consider the following hazardous work activities: • work which exposes children to physical, emotional or sexual abuse. • work underground, under water, at dangerous heights or in confined spaces. • **work with dangerous machinery**, equipment and tools, or which involves the manual handling or transport of heavy loads. • work in an unhealthy environment which may, for example, expose children to hazardous substances, agents or processes, or to temperatures, noise levels, or vibrations damaging to their health. • work under particularly difficult conditions such as work for long hours or during the night or work where the child is unreasonably confined to the premises of the employer.	

Child labour has been previously recognised as a public health issue in the South Asia region where the International Labour Organization (ILO), estimates that child labour (aged 5–17 years old) varies from 5.8 million in India to 2 million in Nepal [11, 12].

With an estimated 1,776,097 children working (of whom 60.14% engaged in hazardous child work), Bangladesh has the second highest burden of child labour in the South Asia region (4.4%) [13]. In recent years, the child labour burden and its devasting individual and social welfare consequences have influenced the Government of Bangladesh to align its legal framework with international standards by ratifying: i) the ILO Convention on the Worst Forms of Child Labour (1999, No. 182) ii) the ILO’s Minimum Age Convention (1973, No 138), iii) the Protocol to the Forced Labour Convention, and by revising the National Plan of Action to Eliminate Child Labour by 2025 [14, 15].

The Bangladesh Labour Act sets the minimum working age at 14 years old and restricts hazardous work for those below 18 years old; in the Act. hazardous work is defined as a work’ that is “likely to hamper child health, safety and moral development, this includes working with dangerous machines that are harmful to their physical and mental development” [16, 17]. Sections 24 and 25 of the Bangladesh Factories Act prohibit children from working around moving machinery or hazardous equipment, such as circular saws, plate-printing machines, and power-driven machines. Young workers operating such machinery must receive proper training, be informed about the dangers, and work under supervision to prevent injuries [18]. Any form of work that encompass hazardous activities is considered the worst forms of child labour (Table 1)**.**

The government also published and updated a list of activities/processes/sectors (e.g. garment) that are officially prohibited for use among children under 18 years old [17–19]. Nonetheless, currently, 95 percent of child labour in Bangladesh is estimated to occur in the informal sector, which falls outside government regulations [14]. With a growing trend of hazardous child labour in urban and semi-urban areas [14, 20]. In these areas, children have been observed working with dangerous machinery, long hours, and harmful conditions many of which are connected to the global supply chain of products [21].

Over recent years, substantial evidence on child labour in the informal sector has highlighted poverty as an overarching driver, compounded by its interplay with proximal and distal contextual factors [14, 20, 22, 23]. Proximal factors include barriers to education, such as hidden schooling costs, poor-quality education, parental illiteracy, and the perception that education offers limited economic returns [20]. Family health crises, such as adult illness, death, disability, or injury, further exacerbate this issue by increasing medical expenses, often forcing children to work to compensate for lost household income [22].

Distal factors include rural-to-urban migration driven by limited opportunities in rural areas and environmental stressors, alongside a demand for unskilled, cheap labour, which creates avenues for exploitation [23]. The absence of effective regulation further enables harmful labour practices to persist unchecked.

Despite documentation of the factors driving child labour, there remains a significant gap in evidence and health data specifically addressing children employed in the informal sector. As the deadline for the Sustainable Development Goal (SDG) to end child labour is upon us, it is paramount to document the health impacts of child labour and highlight this neglected public health issue. Such evidence is critical to inform targeted interventions and policies aimed at protecting vulnerable children and breaking the cycle of exploitation. The present study aims to contribute to this knowledge gap by presenting a retrospective multiyear analysis (2014–2023) of medical data collected by MSF occupational health clinics among children working in the informal sector in a peri-urban area of Dhaka and highlight this largely invisible workforce. To the best of our knowledge, this study is the first of its kind to systematically collect and analyse data emerging from occupational health clinics, disaggregated by sex, age, and place of work. By doing so, it provides robust evidence on the impact of child labour on health, thereby contributing to the global effort to inform elimination of child labour and its exploitation forms through data-driven insights.

## Methods

### Study setting

Kamrangirchar, located on the Buriganga River, is one of Dhaka’s largest peri-urban areas, with a population of 440,000 concentrated in an area of four square kilometres [24, 25]. It is estimated that Kamrangirchar has over 1,000 informal factories, including those mainly in the metal, garment, and plastics sectors, along with tanneries that relocated to Savar in April 2017 [26, 27]. In Kamrangirchar, MSF obtained approval from the Government of Bangladesh to implement occupational health clinics. MSF subsequently started two occupational health clinics in 2013 to provide medical care and psychological support to workers from the informal factory sector. The MSF team set up agreements with factory owners/managers across tannery, garment, metal and plastics sectors allowing factory workers to attend health clinics during their working hours. Workers from these registered factories could access MSF occupational health services. However, workers from unregistered factories (no formal agreement with MSF) could also access emergency services and first aid for their injuries.

### Study design and participants

In this retrospective study, we used anonymised primary health care records from the MSF occupational health clinics Excel database. The occupational work-related diseases listed by the US Department of Labor, Occupational Safety and Health (OSH) classification and in the International Classification of Diseases, work-related diseases (ICD 10) list were used in the diagnosis of workers who attended the clinics [28]. The following core variables were chosen for inclusion in this study as they were collected across the whole study period: date of consultation, age, sex, visit status (new/follow-up), residence inside a factory, type of factory they worked in, if they were working with machines inside the factory, nutritional, and mental health status, type and mechanism of injury and diagnosis. The occupational health clinicians had both checklists and protocols and relied on ICD- 10 definitions for their diagnoses. Multiple health conditions were recorded, where relevant, but only the primary diagnoses were included in this study.

Our population consisted of children and adolescents under 18 years presenting to MSF occupational health clinics between February 2014 to December 2023. The occupational health clinics remained consistently open throughout this period although the number of consultations dropped considerably in 2020 due to COVID- 19 and returned to pre-COVID- 19 levels by March 2021. The mental health dataset was a separate Excel database and a subset of the main occupational health dataset and consisted of consultation data between September 2018 to December 2023.

### Variable definitions

New consultations were defined as patients who came for the first time for a consultation. Follow-up consultations were defined as patients who came for subsequent appointments (scheduled or walk-ins) to reassess and manage the patient's ongoing occupational health condition.

Nutritional status among workers less than 18 years was assessed using Body Mass Index (BMI). The World Health Organization growth standards were used to define BMI-for-age score, with adjustments made by MSF to ensure suitability for the study population [29]. No further adjustments for sex or ethnic differences were made, as population-specific references were limited. Those with a BMI-for-age more than two standard deviations less than the median for their age group were classified as malnourished. Other measures of nutritional status such as anaemia or stunting were not part of routine data collection for occupational health patients.

Work-related morbidity refers to any physical or psychological health conditions that are directly caused or exacerbated by the patient's job or working conditions. This includes illnesses, chronic conditions, injuries, and psychological distress that arise from factors such as repetitive strain, exposure to hazardous materials, workplace accidents, or stressful work environments. In our analysis, a"yes"for work-related morbidity indicates a decision made by an occupational health doctor that the individual has a health condition directly caused or exacerbated by their job or working conditions, while"no"indicates that the individual does not have any such work-related health conditions.

Living inside the factory was defined as children reporting the factory as their primary residence, such as where they sleep. This variable is important as it might expose children to additional environmental work hazards (e.g., noise, chemicals), while also limiting mobility and social interactions. Moreover, workers who live on-site may be subject to extended working hours, increasing the risk of overexertion, fatigue, and occupational accidents.

### Data analysis

Descriptive analysis of the occupational health data among children under 18 years old was performed. The analysis of the occupational health data primarily focused on new consultations, as they provided an indication of the burden of occupational morbidities. The analysis was stratified by sex and age group (comparing those under 14 years to those aged 14-under 18 years). In addition, the type of factory/sector where children were employed and if children reported working with machines.Working with machinery was used as a proxy for hazardous work, consistent with the Bangladesh Labour Act, due to its known significant risks to children's safety, including serious injuries such as amputations, burns, and crushing. These risks are both direct and easily identifiable, making machinery a practical and effective indicator for assessing hazardous work.

Another hazardous proxy considered was the type of factory where children worked, particularly those that fit the list of prohibited industries outlined by the Bangladesh government (e.g. garments, battery, chemical, leather, tanneries, plastic, rubber) [18, 19].

We choose those two age groups, since the Bangladesh Labour Act, 2006 (and Amendments) defines a child as anyone under the age of 14 years and prohibits their employment in any establishment (Sect. 34) and prohibits the employment of adolescents (aged 14–17) in hazardous work (Sect. 39). Chi-squared and fisher’s exact tests were used to check for differences in proportions across categorical variables. The analysis was performed using R Studio software (R v 4. 3.2) [30].

## Results

### Demographics

Over the study period, there were a total of 10,200 occupational health consultations among children aged under 18 years. Of the 10,200 consultations, there were 4945 new consultations, 3895 were follow-up consultations, and consultation status was missing for 1360 records (Table 2). There was an average of 494 new consultations among children every year with a peak of 967 in 2018.

Among the new consultations, 38% were among children aged under 14 years (the youngest child was five years), the mean age was 14.7 years (standard deviation of 2.02 years) and 61% were male. Eighty-five per cent of the children reported living outside the factory (Table 2). Overall, male children were more likely to live inside the factory compared to female children (23% vs 1%, *p* < 0.001) (see additional file 1).

**Table 2:** Demographic characteristics of new occupational health patients < 18 years, MSF clinics, Dhaka, 2014–2023

Characteristic	Overall, *N* = 4,945^a^	< 14, *N* = 1,882^a^	> = 14–17, *N* = 3,063^a^	*p*-value^2^
Sex				0.004
Female	1,950 (39%)	694 (37%)	1,256 (41%)	
Male	2,995 (61%)	1,188 (63%)	1,807 (59%)	
Residence				< 0.001
Lives outside factory	3,570 (85%)	1,417 (88%)	2,153 (84%)	
Lives inside factory	611 (15%)	189 (12%)	422 (16%)	
Unknown	764	276	488	

^a^n (%)

^2^Pearson's Chi-squared test

### Place of work

Almost a third (32%) of new consultations were among children who worked in garment factories, followed by plastics (30%) and metal (21%) factories (Table 3). Female children attending new consultations were predominantly employed in plastics and garment factories, whereas male children were more frequently employed in garment and metal factories (Table 3). Similar proportions of children aged < 14 and 14–17 years worked in the various factory types (Additional file 1). Ninety six percent of children irrespective of age group or sex reported operating machinery as part of their work (Additional file 1). Most children reported operating machinery across all factory types (Additional file 1).

**Table 3:** Place of work among new occupational health patients < 18 years by sex MSF clinics, Dhaka, 2014–2023

**Characteristic**	**Overall**, *N* = 4,945^a^	**Female**, *N* = 1,950^a^	**Male**, *N* = 2,995^a^
Type of factory			
Garment	1,575 (32%)	724 (37%)	851 (29%)
Plastics	1,483 (30%)	750 (39%)	733 (25%)
Metal	1,033 (21%)	187 (9.6%)	846 (28%)
Leather	195 (4.0%)	88 (4.5%)	107 (3.6%)
Embroidery	181 (3.7%)	69 (3.6%)	112 (3.8%)
Tannery	141 (2.9%)	16 (0.8%)	125 (4.2%)
Rubber	148 (3.0%)	42 (2.2%)	106 (3.6%)
Other	136 (2.8%)	65 (3.3%)	71 (2.4%)
Chemical	26 (0.5%)	1 (< 0.1%)	25 (0.8%)
Battery	1 (< 0.1%)	1 (< 0.1%)	0 (0%)
Unknown	26	7	19

^a^n (%)

### Morbidities

Musculoskeletal and dermatology (e.g., fungal infection, mainly scabies) diagnoses accounted for almost half of all new consultations among children, with similar proportions across age group and sex (Table 4 and additional file 1). In addition, no major differences were observed in conditions whether a child lived inside or outside a factory (Additional file 1). Work related injuries accounted for 7.5% of all new consultations among children and with no difference by age group. However, male children had a higher proportion of injuries (11%) compared to female children (2.5%) (Additional file 1). The majority (83%) of the diagnoses irrespective of age group or sex were suspected as being work-related. Sixty-three per cent of under 14 years were found to be malnourished compared to 40% of 14–17 years and similarly a higher proportion of male children compared to female (Additional file 1).

**Table 4:** Primary diagnoses among new occupational health patients < 18 years, MSF clinics, Dhaka, 2014–2023

**Characteristic**	**Overall**, *N* = 4,945^a^	** < 14**, *N* = 1,882^a^	** > = 14–17**, *N* = 3,063^a^	***p*-value**^2^
Primary diagnosis				
Musculoskeletal	1,197 (26%)	455 (26%)	742 (26%)	
Dermatology	907 (20%)	302 (17%)	605 (22%)	
Respiratory	632 (14%)	281 (16%)	351 (12%)	
Gastro-intestinal (GI)	550 (12%)	184 (11%)	366 (13%)	
Injury	339 (7.5%)	134 (7.7%)	205 (7.3%)	
ENTDEH (Ear, Nose, Throat, Dental, Eyes and Head)	419 (9.2%)	163 (9.4%)	256 (9.1%)	
Others	167 (3.7%)	75 (4.3%)	92 (3.3%)	
Other Chronic Condition	128 (2.8%)	79 (4.5%)	49 (1.7%)	
SRH (Sexual & Reproductive Health)	131 (2.9%)	40 (2.3%)	91 (3.2%)	
Infectious Disease	17 (0.4%)	5 (0.3%)	12 (0.4%)	
Cardiovascular	26 (0.6%)	6 (0.3%)	20 (0.7%)	
Urinary	11 (0.2%)	4 (0.2%)	7 (0.2%)	
Hematology	7 (0.2%)	2 (0.1%)	5 (0.2%)	
Neurological Disorder	13 (0.3%)	6 (0.3%)	7 (0.2%)	
Mental Health	3 (< 0.1%)	0 (0%)	3 (0.1%)	
Non-Communicable Disease	2 (< 0.1%)	1 (< 0.1%)	1 (< 0.1%)	
Unknown	396	145	251	
Suspected occupational condition				0.6
Work-related	2,971 (83%)	1,138 (84%)	1,833 (83%)	
Non-work related	588 (17%)	219 (16%)	369 (17%)	
Unknown	1,386	525	861	
Nutrition status				< 0.001
Normal (BMI for age > − 2SD)	2,217 (51%)	607 (37%)	1,610 (60%)	
Malnourished (BMI for age < − 2SD)	2,121 (49%)	1,050 (63%)	1,071 (40%)	
Unknown	607	225	382	

^a^n (%)

^2^Pearson's Chi-squared test

Among the 51 mental health outcomes recorded between September 2018 to December 2023, the primary mental health disorders diagnosed were mood-related (86%) and behavioural symptoms (10%), with most patients being male (55%) and aged 14–17 years (80%) (Table 5). Key precipitating events included family disruptions, domestic violence, and socio-economic issues, reported by half of the patients. Domestic violence was more prevalent among females (17%) compared to males (4%), (Table 5).

**Table 5:** Mental health characteristics of new occupational health patients < 18 years by sex, MSF clinics, Dhaka, 2014–2023

**Characteristic**	**Overall**, *N* = 51^a^	**Female**, *N* = 23	**Male**, *N* = 28	***p*-value**^2^
**Age**				> 0.9
< 14	10 (20%)	4 (17%)	6 (21%)	
> = 14–17	41 (80%)	19 (83%)	22 (79%)	
**Type of mental health disorder**				> 0.9
Mood related problems	43 (86%)	21 (91%)	22 (81%)	
Behavior related symptoms	5 (10%)	2 (9%)	3 (11%)	
Neuro-psychiatric related symptoms	1 (2%)	0 (0%)	1 (4%)	
Social Functioning	1 (2%)	0 (0%)	1 (4%)	
Unknown	1	0	1	
**Precipitating event**				0.2
Disruption of family & relationships	12 (26%)	7 (30%)	5 (22%)	
Domestic discord & family violence	5 (11%)	4 (17%)	1 (4%)	
Socio-economic functioning	5 (11%)	3 (13%)	2 (9%)	
Events related to abuse during detention	2 (4%)	0 (0%)	2 (9%)	
Medical illness-related	2 (4%)	0 (0%)	2 (9%)	
Events related to violence	1 (2%)	1 (4%)	0 (0%)	
Sexual trauma or abuse	1 (2%)	1 (4%)	0 (0%)	
Neuro-psychiatric related	1 (2%)	1 (4%)	0 (0%)	
Others	17 (37%)	6 (26%)	11 (48%)	
Unknown	5	0	5	

^a^n (%)

^2^Fisher's exact test

Among the 182 injuries diagnosed in new consultations across the study period, the majority occurred in patients reporting working in metal factories (65%), followed by plastics (20%) and garments (7%) (Table 6). The proportions of injuries by factory type were similar across age groups (Additional file 1). There were differences by sex such that there were higher proportions of male children with injuries overall and particularly among those working in metal factories and higher proportions of female children with injuries working in plastics factories (Table 6). Cuts/lacerations accounted for over 60% of the recorded injuries and over 70% were due to patients being struck by an object (Table 6).

**Table 6:** Injury characteristics among new occupational health patients < 18 years by sex, MSF clinics, Dhaka, 2014–2023

Characteristic	Overall, *N* = 182^a^	Female, *N* = 26^a^	Male, *N* = 156^a^	*p*-value^2^
Type of factory				< 0.001
Metal	119 (65%)	7 (27%)	112 (72%)	
Plastics	36 (20%)	12 (46%)	24 (15%)	
Garment	13 (7.1%)	3 (12%)	10 (6.4%)	
Embroidery	7 (3.8%)	0 (0%)	7 (4.5%)	
Other	6 (3.3%)	4 (15%)	2 (1.3%)	
Leather	1 (0.5%)	0 (0%)	1 (0.6%)	
Rubber	0 (0%)	0 (0%)	0 (0%)	
Tannery	0 (0%)	0 (0%)	0 (0%)	
Type of injury				0.4
Cut/laceration	100 (61%)	14 (61%)	86 (61%)	
Other	29 (18%)	5 (22%)	24 (17%)	
Crushing injury	14 (8.6%)	4 (17%)	10 (7.1%)	
Burn	10 (6.1%)	0 (0%)	10 (7.1%)	
Abrasion	8 (4.9%)	0 (0%)	8 (5.7%)	
Amputation	2 (1.2%)	0 (0%)	2 (1.4%)	
Broken bone	0 (0%)	0 (0%)	0 (0%)	
Bruise	0 (0%)	0 (0%)	0 (0%)	
Concussion	0 (0%)	0 (0%)	0 (0%)	
Unknown	19	3	16	
Mechanism of injury				0.3
Struck	130 (72%)	18 (69%)	112 (73%)	
Other	26 (14%)	3 (12%)	23 (15%)	
Fall	11 (6.1%)	4 (15%)	7 (4.5%)	
Burn	6 (3.3%)	0 (0%)	6 (3.9%)	
Caught in between	7 (3.9%)	1 (3.8%)	6 (3.9%)	
Unknown	2	0	2	

^a^n (%)

^2^Fisher's exact test

## Discussion

Globally, one in ten children are engaged in child labour, and in some countries one in four are engaged in labour that is considered harmful to their health and development [31, 32]. Evidence suggests that hazardous child labour can have devastating consequences that directly endangers a child’s health, safety, and moral development [5]. It can result in disability, ill health and psychological damage, which prevents children of all ages from going to school, takes them away from their families, uses up time for play and recreation in the company of their peers, and causes significant short- and long-term harm [9, 33]. A review of 25 studies in low- and middle-income countries further identifies child labour as being linked to adverse health outcomes such as poor growth, malnutrition, higher incidence of infectious diseases, behavioural and emotional disorders, and decreased coping abilities [5]. Far from having a positive effect, it impedes children’s growth and development, as well as perpetuates a cycle of poverty for the children involved, their families and communities.

To our knowledge, our analysis is the first to generate robust medical data emerging from a multi-year (2014–2023) occupational health intervention, in a peri-urban area of Bangladesh where the informal economy is prevalent. This study gives visibility to a heterogenous, neglected population and adds important evidence on health among child workers. Findings show that children as young as five years have been engaged in hazardous tasks (e.g. labour involving machinery) and are operating in all sectors currently forbidden in the country (e.g. garments, battery, chemical, leather, tanneries, plastic, rubber). Any children engaged in these sectors/tasks are deemed to be engaged in hazardous child labour classified as the worst form of child labour. The young age of child labourers identified in this study is in line with the National Child Labour Survey conducted in Bangladesh in 2022 and similar in studies from Pakistan and India [13, 34, 35].

In our study, almost 15% of the children reported they were living inside the factory where they were employed. Living inside the factories was more frequent among male children across both age groups (under 14 and under 18 years old) compared to girls. Living and working in a hazardous context might add additional health burden and social alienation for children, and likely expose children to additional risks which include, physical, emotional or sexual abuse [5, 36].

We observed concerning patterns of morbidity with both age groups experiencing a high burden of musculoskeletal, and dermatological conditions (e.g. fungal infection; scabies). A similar high burden of these conditions was reported from studies in child workers in India, Pakistan, Bangladesh, and Brazil [5, 37–39].

In addition, in our study almost 50% of children were malnourished. High levels of malnutrition have been previously reported in a study among 100 child workers (aged 5–17 years) in Dhaka [40]. Other researchers have highlighted disruptions in regular meal patterns among child labourers, with many starting their workdays hungry and struggling to find time for proper meals during long shifts [22]. Similarly, studies in India found that malnutrition was likely driven by long working hours and inadequate nutrition [41]. In Pakistan, food insecurity and poor nutritional status were prevalent among child labourers, with high rates of stunting, wasting, and inadequate food intake, particularly in agriculture and migrant labour sectors [42]. Similarly, in Malawi, malnutrition was a concern among children working in the informal sector, including tobacco farming [43].

Almost all children in this study reported working with machines in sectors forbidden by current Bangladesh legislation, with 7.5% of children experiencing injuries with cuts or laceration being the most frequently documented injury type. Previous surveys in Bangladesh reported 18.5% of children engaged in hazardous work, with a significant portion facing injuries and abuse [44]. Another study conducted in the slums/suburbs of Dhaka that looked at the pattern of injuries among children of urban slum dwellers in Dhaka City, found that occupational injury was the third highest cause of injuries among surveyed children and second among 10–15 years age group [45]. The CLARISSA study, conducted in collaboration with child labourers in Dhaka, documented widespread injuries among children working in the leather industry, including cuts, wounds, skin problems. These injuries were exacerbated by the absence of adequate safety measures in workplaces where children are employed [22]. Outside of Bangladesh, other authors have examined the relationship between working conditions and the incidence of injuries among Syrian refugee children in Lebanon [46]. This study revealed that factors such as multiple jobs, work pressure, and physical abuse increased the likelihood of injuries, with notable sex differences.

Children might be more vulnerable than adults to workplace hazards. Previous MSF consultations with parents and caregivers highlighted the profound sorrow experienced by parents witnessing their children being injured. *"It is heartbreaking. There are times when my son suffers from cuts and injuries on his hands… Frequent cuts on their legs and injuries on their lips, along with abrasions on their chests are a common occurrence. Witnessing their pain and injuries fills us with sorrow, but unfortunately, we are helpless to prevent them."*—Caregiver of a young factory worker [1]. Mental health has also emerged as a concern in both age groups, with mood-related disorders and behavioural symptoms being most diagnosed. The prevalence of these mental health issues is consistent with findings from other studies on child labour, emphasizing the psychological impact of stressful and abusive work environments [47].

In our study contributing factors include family disruptions, domestic violence, and socio-economic challenges, with domestic violence particularly affecting female children. This intersection of risks from both work and home environments underscores their combined influence on mental health outcomes among child workers. This aligns with evidence from Jordan, Lebanon, Ethiopia, and Pakistan, where child labourers face emotional disturbances, anxiety, depression, and significant difficulties in coping with abuse, loneliness, and stress from their work and family environments exacerbating mental health challenges [5].

In our study we identified specific sex and age vulnerabilities. For instance, more male children compared to female reported living inside the factories, experiencing injuries, and presenting with poor nutritional status. On the other hand, females reported more musculoskeletal and dermatological conditions, and overall female children represented a lower proportion of consultations. However, these differences could be explained as a disparity in access or by a differential mobility across the two sexes and by the type of factory with whom MSF set up an agreement. Regarding age-related disparities, we documented that prevalence of malnutrition was higher among children under 14 years compared to children aged 14–17 years old.

The ILO, Decent Work Country Program (DWCP) 2022–2026 framework for Bangladesh, delineated some aspects to improve health and safety for children working in the informal sector [48]. However, our data shows that children across different age groups experience different vulnerabilities that will require a dedicated framework [48]. Current gaps contribute to continued exploitation of children and delay elimination of child labour and its worst form manifestations. Furthermore, ILO’s definitions and metrics on child labour overlook key aspects of the United Nations Convention on the Rights of the Child (UNCRC), failing to consider the structural and intersectional factors that drive social exclusion and inequality, creating a homogeneous group that might further increase children’s inequalities. Current policy overlooks the fact that neoliberal, unregulated supply demands are the key harmful global practices, and child labour is often a coping mechanism to those dysfunctional demands. This ultimately place the burden on individuals and communities instead of addressing the root causes. A radical reframing is needed, with a particular emphasis on children as active agent of change.

Specific short-term harm reduction initiatives (e.g. tetanus immunization, training, protective equipment, clear safety instruction, shorter hours, light tasks which are less exposed to chemicals, heavy machinery, noise etc.) for older children (above 14 years old) could mitigate several types of consequences of worst forms of work. However, besides the immediate short-term interventions, there is a clear need for initiatives to cooperate with child welfare bodies and civil society organizations towards removing children who are too young to work legally and protect older children from hazardous and undignified conditions in the long term [49].

This will require adaptation of existing child labour law to encompass a public health multi-sector approach including social-case management, integration into learning and economic assistance for parents and caregivers, along with awareness campaigns related to child labour. Similarly, referral to specialised mental health services and counselling for children experiencing exploitation; rehabilitation services for work-related impairments and to essential services such as education, and child protection.

Currently lack of occupational health care and nutrition programmes in places where child labour occurs further harms children’s wellbeing, and limits opportunities for referral. This lack of services also presents a barrier to generating solid evidence on the impact of child labour and the worst forms of child labour on a child’s health to inform targeted and effective interventions along with public health awareness campaigns.

Overall, greater attention needs to be paid to the tens of thousands of small businesses which exist in the shadow economy, as the informal sector is where the worst forms of child labour are found and to the different economic drivers that push children into a context of exploitation [50].

### Strengths and limitations

There are several limitations and strengths of this study, Firstly, there is a lack of qualitative data to explore children’s perspectives and the impact of labour on general and mental health wellbeing. A lack of data on variables of work exposure that could be used as predictor of morbidities, nutritional status or injuries and lack of a control group. The data is limited by its treatment-focused approach, which may not fully capture the breadth of occupational hazards, particularly those related to machinery use. A limited exploration of varied machinery types restricts understanding of diverse occupational hazards. Furthermore, the study lacked data on education, hours of work, salary received by children that could have helped to understand the severity of exploitation. Data are related to children who had access to the occupational clinic but is likely that other severe and mild morbidities have not been captured. However, only a surveillance system inside the factories could monitor and capture the extent of morbidities. In addition, this was a retrospective analysis of occupational health service data and therefore may not fully capture the experiences of child workers who did not seek medical care or who were employed in factories not registered with MSF. Another limitation is the absence of data capturing the severity of injuries, limiting our understanding of immediate and long-term disability consequences. In addition, we do not have information on the number of follow-up consultations related to the morbidity diagnosed in the first visit due to a lack of analysis of follow-up consultation data. Mental health data were limited; thus, we might have incomplete information on the extent of the impact of hazardous work on children.

However, important strengths of this study are that data have been collected by trained staff on occupational health and have been consistent over time. Morbidities observed are largely corroborated by existing literature on health among child labourers. Additional strengths include the large number of diverse patients. The study provides insights into the diagnosis of mental health issues among child workers, shedding light on the often-overlooked psychological challenges stemming from their exposure to stressful and abusive work environments. Finally, the study provides reliable data on children under 14 years, data rarely available that are essential to design targeted interventions.

## Conclusion

As the deadline for the Sustainable Development Goal to end child labour is upon us, it is paramount to document the impact of child labour on health and formulate public health solutions. Findings suggest that children face hazardous realities, engaged in the worst form of labour, bearing important morbidity and injury burden. Poor nutritional status further compounds an already fragile health status.

Children are engaged in all the categories of the informal sector, however despite their substantial contribution to the economy, they remain a largely invisible workforce, at the edge of society, excluded from essential social and legal protection framework thus hampering children’s rights and health. Poor incorporation of the UNCRC principles in ILO and labour regulations for the informal sector ultimately contribute to child exploitation, suffering, and fueling a society of poverty and exclusion. 

Further research is essential to explore the intersectional dimensions of child labour—including sex, age, disability, family circumstances, migration status, and the types of harmful practices to which children are exposed— to identify specific vulnerabilities. Engaging in rights-based lived experience research, including community-based approaches and arts-based methods, in collaboration with communities and children, can provide deeper contextual insights into the complex dimensions of child labour, helping to amplify the voices of those directly affected and ensuring their perspectives are central to the development of effective interventions in this and similar contexts.

Finally, this study advocates for integrated efforts across the education, protection, child rights, research, and occupational health sectors, involving governments, factory owners, families, non-governmental organisations, and the international community, with a strong emphasis on direct engagement with children to develop sustainable, context-specific solutions for addressing this preventable public health wound.

## Supplementary Information

Additional file 1. Includes eight supporting tables : (1) Residence of new occupational health patients <18 years by sex, MSF clinics, Dhaka, 2014-2023; (2) Place of work of new occupational health patients < 18 years by age, MSF clinics, Dhaka, 2014-2023 (3) Machinery operation among new occupational health patients < 18 years by sex, MSF clinics, Dhaka, 2014-2023; (4) Machinery operation among new occupational health patients < 18 years by age, MSF clinics, Dhaka, 2014-2023; (5) Machinery operation among new occupational health patients < 18 years by place of work, MSF clinics, Dhaka, 2014-2023; (6) Primary diagnoses among new occupational health patients <18 years by sex, MSF clinics, Dhaka, 2014-2023: (7) Primary diagnoses among new occupational health patients <18 years by place of residence, MSF clinics, Dhaka, 2014-2023 and (8) Injury characteristics among new occupational health patients < 18 years by age, MSF clinics, Dhaka, 2014-2023

## Data Availability

The datasets supporting the conclusions of this article are available on request in accordance with MSF’s data sharing policy. Requests for access to data should be made to oca.research@london.msf.org.
