# Supplementary material for: A Public health wound: health and work among children engaged in the worst forms of child labour in the informal sector in Dhaka, Bangladesh: a retrospective analysis of Médecins Sans Frontières occupational health data from 2014 to 2023
Source: BMC Public Health. 2025 Apr 15;25:1420. doi: 10.1186/s12889-025-22483-z (PMC12001455; doi:10.1186/s12889-025-22483-z)
Supplement: Supplementary file 1 — Additional file 1. Includes eight supporting tables : (1) Residence of new occupational health patients <18 years by sex, MSF clinics, Dhaka, 2014-2023; (2) Place of work of new occupational health patients < 18 years by age, MSF clinics, Dhaka, 2014-2023 (3) Machinery operation among new occupational health patients < 18 years by sex, MSF clinics, Dhaka, 2014-2023; (4) Machinery operation among new occupational health patients < 18 years by age, MSF clinics, Dhaka, 2014-2023; (5) Machinery operation among new occupational health patients < 18 years by place of work, MSF clinics, Dhaka, 2014-2023; (6) Primary diagnoses among new occupational health patients <18 years by sex, MSF clinics, Dhaka, 2014-2023: (7) Primary diagnoses among new occupational health patients <18 years by place of residence, MSF clinics, Dhaka, 2014-2023 and (8) Injury characteristics among new occupational health patients < 18 years by age, MSF clinics, Dhaka, 2014-2023 [file 12889_2025_22483_MOESM1_ESM.docx]

Table 1: Residence of new occupational health patients <18 years by sex, MSF clinics, Dhaka, 2014-2023

| **Characteristic** | **Overall,** N = 4,945^1^ | **Female,** N = 1,950^1^ | **Male,** N = 2,995^1^ | **p-value^2^** |
| --- | --- | --- | --- | --- |
| Residence |  |  |  | <0.001 |
| Lives outside factory | 3,570 (85%) | 1,623 (99%) | 1,947 (77%) |  |
| Lives inside factory | 611 (15%) | 22 (1.3%) | 589 (23%) |  |
| Unknown | 764 | 305 | 459 |  |
| ^1^n (%) | | | | |
| ^2^Pearson's Chi-squared test | | | | |

Table 2: Place of work of new occupational health patients <18 years by age, MSF clinics, Dhaka, 2014-2023

| **Characteristic** | **Overall**, N = 4,945^1^ | **<14**, N = 1,882^1^ | **>=14-17**, N = 3,063^1^ |
| --- | --- | --- | --- |
| Type of factory |  |  |  |
| Garment | 1,575 (32%) | 565 (30%) | 1,010 (33%) |
| Plastics | 1,483 (30%) | 563 (30%) | 920 (30%) |
| Metal | 1,033 (21%) | 423 (23%) | 610 (20%) |
| Leather | 195 (4.0%) | 48 (2.6%) | 147 (4.8%) |
| Embroidery | 181 (3.7%) | 45 (2.4%) | 136 (4.5%) |
| Tannery | 141 (2.9%) | 68 (3.6%) | 73 (2.4%) |
| Rubber | 148 (3.0%) | 79 (4.2%) | 69 (2.3%) |
| Other | 136 (2.8%) | 72 (3.9%) | 64 (2.1%) |
| Chemical | 26 (0.5%) | 6 (0.3%) | 20 (0.7%) |
| Battery | 1 (<0.1%) | 1 (<0.1%) | 0 (0%) |
| Unknown | 26 | 12 | 14 |
| ^1^n (%) | | | |

Table 3: Machinery operation among new occupational health patients <18 years by sex, MSF clinics, Dhaka, 2014-2023

| **Characteristic** | **Overall**, N = 4,945^1^ | **Female**, N = 1,950^1^ | **Male**, N = 2,995^1^ | **p-value**^2^ |
| --- | --- | --- | --- | --- |
| Task in factory |  |  |  | 0.006 |
| Operates machine | 4,760 (96%) | 1,895 (97%) | 2,865 (96%) |  |
| Does not operate machinery | 185 (3.7%) | 55 (2.8%) | 130 (4.3%) |  |
| ^1^n (%) | | | | |
| ^2^Pearson's Chi-squared test | | | | |

Table 4: Machinery operation among new occupational health patients <18 years by age, MSF clinics, Dhaka, 2014-2023

| **Characteristic** | **Overall**, N = 4,945^1^ | **<14**, N = 1,882^1^ | **>=14-17**, N = 3,063^1^ | **p-value**^2^ |
| --- | --- | --- | --- | --- |
| Task in factory |  |  |  | 0.024 |
| Operates machine | 4,760 (96%) | 1,797 (95%) | 2,963 (97%) |  |
| Does not operate machinery | 185 (3.7%) | 85 (4.5%) | 100 (3.3%) |  |
| ^1^n (%)  ^2^Pearson's Chi-squared test | | | | |

| Table 5: Machinery operation among new occupational health patients <18 years by place of work, MSF clinics, Dhaka, 2014-2023   \| **Characteristic** \| **Operates machine**, N = 4,760^1^ \| **Does not operate machinery**, N = 185^1^ \| \| --- \| --- \| --- \| \| Type of factory \|  \|  \| \| Garment \| 1,528 (97%) \| 47 (3.0%) \| \| Plastics \| 1,424 (96%) \| 59 (4.0%) \| \| Metal \| 992 (96%) \| 41 (4.0%) \| \| Leather \| 186 (95%) \| 9 (4.6%) \| \| Embroidery \| 180 (99%) \| 1 (0.6%) \| \| Tannery \| 128 (91%) \| 13 (9.2%) \| \| Rubber \| 143 (97%) \| 5 (3.4%) \| \| Other \| 131 (96%) \| 5 (3.7%) \| \| Chemical \| 26 (100%) \| 0 (0%) \| \| Battery \| 1 (100%) \| 0 (0%) \| \| Unknown \| 21 \| 5 \| \| ^1^n (%) \| \| \| |
| --- | --- | --- | --- | --- | --- | --- | --- | --- | --- | --- | --- | --- | --- | --- | --- | --- | --- | --- | --- | --- | --- | --- | --- | --- | --- | --- | --- | --- | --- | --- | --- | --- | --- | --- | --- | --- | --- | --- | --- | --- | --- | --- |

Table 6: Primary diagnoses among new occupational health patients <18 years by sex, MSF clinics, Dhaka, 2014-2023

| **Characteristic** | **Overall**, N = 4,945^1^ | **Female**, N = 1,950^1^ | **Male**, N = 2,995^1^ | **p-value**^2^ |
| --- | --- | --- | --- | --- |
| Primary diagnosis |  |  |  |  |
| Musculoskeletal | 1,197 (26%) | 507 (29%) | 690 (25%) |  |
| Dermatology | 907 (20%) | 308 (17%) | 599 (22%) |  |
| Respiratory | 632 (14%) | 205 (12%) | 427 (15%) |  |
| Gastro-intestinal (GI) | 550 (12%) | 233 (13%) | 317 (11%) |  |
| Injury | 339 (7.5%) | 44 (2.5%) | 295 (11%) |  |
| ENTDEH (Ear, Nose, Throat, Dental, Eyes and Head) | 419 (9.2%) | 192 (11%) | 227 (8.2%) |  |
| Others | 167 (3.7%) | 77 (4.4%) | 90 (3.2%) |  |
| Other Chronic Condition | 128 (2.8%) | 37 (2.1%) | 91 (3.3%) |  |
| SRH (Sexual & Reproductive Health) | 131 (2.9%) | 129 (7.3%) | 2 (<0.1%) |  |
| Infectious Disease | 17 (0.4%) | 4 (0.2%) | 13 (0.5%) |  |
| Cardiovascular | 26 (0.6%) | 11 (0.6%) | 15 (0.5%) |  |
| Urinary | 11 (0.2%) | 6 (0.3%) | 5 (0.2%) |  |
| Hematology | 7 (0.2%) | 6 (0.3%) | 1 (<0.1%) |  |
| Neurological Disorder | 13 (0.3%) | 7 (0.4%) | 6 (0.2%) |  |
| Mental Health | 3 (<0.1%) | 0 (0%) | 3 (0.1%) |  |
| Non-Communicable Disease | 2 (<0.1%) | 0 (0%) | 2 (<0.1%) |  |
| Unknown | 396 | 184 | 212 |  |
| Suspected occupational condition |  |  |  | 0.026 |
| Work-related | 2,971 (83%) | 1,167 (82%) | 1,804 (85%) |  |
| Non-work related | 588 (17%) | 260 (18%) | 328 (15%) |  |
| Unknown | 1,386 | 523 | 863 |  |
| Nutrition status |  |  |  | <0.001 |
| Normal | 2,217 (51%) | 976 (57%) | 1,241 (47%) |  |
| Malnourished | 2,121 (49%) | 737 (43%) | 1,384 (53%) |  |
| Unknown | 607 | 237 | 370 |  |
| ^1^n (%) | | | | |
| ^2^Pearson's Chi-squared test | | | | |

Table 7: Primary diagnoses among new occupational health patients <18 years by place of residence, MSF clinics, Dhaka, 2014-2023

| **Characteristic** | **Overall**, N = 4,181^1^ | **Lives inside factory**, N = 611^1^ | **Lives outside factory**, N = 3,570^1^ |
| --- | --- | --- | --- |
| Primary diagnosis |  |  |  |
| Musculoskeletal | 1,043 (27%) | 149 (27%) | 894 (27%) |
| Dermatology | 743 (19%) | 133 (24%) | 610 (19%) |
| Respiratory | 524 (14%) | 87 (16%) | 437 (13%) |
| Gastro-intestinal (GI) | 432 (11%) | 72 (13%) | 360 (11%) |
| Injury | 306 (7.9%) | 30 (5.3%) | 276 (8.4%) |
| ENTDEH (Ear, Nose, Throat, Dental, Eyes and Head) | 364 (9.4%) | 51 (9.1%) | 313 (9.5%) |
| Others | 155 (4.0%) | 16 (2.9%) | 139 (4.2%) |
| Other Chronic Condition | 107 (2.8%) | 10 (1.8%) | 97 (2.9%) |
| SRH (Sexual & Reproductive Health) | 112 (2.9%) | 1 (0.2%) | 111 (3.4%) |
| Infectious Disease | 16 (0.4%) | 4 (0.7%) | 12 (0.4%) |
| Cardiovascular | 24 (0.6%) | 2 (0.4%) | 22 (0.7%) |
| Urinary | 11 (0.3%) | 2 (0.4%) | 9 (0.3%) |
| Hematology | 5 (0.1%) | 0 (0%) | 5 (0.2%) |
| Neurological Disorder | 10 (0.3%) | 4 (0.7%) | 6 (0.2%) |
| Mental Health | 3 (<0.1%) | 0 (0%) | 3 (<0.1%) |
| Non-Communicable Disease | 2 (<0.1%) | 0 (0%) | 2 (<0.1%) |
| Unknown | 324 | 50 | 274 |

^1^n (%)

Table 8: Injury characteristics among new occupational health patients <18 years by age, MSF clinics, Dhaka, 2014-2023

| **Characteristic** | **Overall**, N = 182^1^ | **<14**, N = 50^1^ | **14-17**, N = 132^1^ | **p-value**^2^ |
| --- | --- | --- | --- | --- |
| Type of factory |  |  |  | 0.9 |
| Metal | 119 (65%) | 35 (70%) | 84 (64%) |  |
| Plastics | 36 (20%) | 8 (16%) | 28 (21%) |  |
| Garment | 13 (7.1%) | 4 (8.0%) | 9 (6.8%) |  |
| Embroidery | 7 (3.8%) | 1 (2.0%) | 6 (4.5%) |  |
| Other | 6 (3.3%) | 2 (4.0%) | 4 (3.0%) |  |
| Leather | 1 (0.5%) | 0 (0%) | 1 (0.8%) |  |
| Rubber | 0 (0%) | 0 (0%) | 0 (0%) |  |
| Tannery | 0 (0%) | 0 (0%) | 0 (0%) |  |
| Type of injury |  |  |  | 0.3 |
| Cut/laceration | 100 (61%) | 28 (70%) | 72 (59%) |  |
| Other | 29 (18%) | 3 (7.5%) | 26 (21%) |  |
| Crushing injury | 14 (8.6%) | 3 (7.5%) | 11 (8.9%) |  |
| Burn | 10 (6.1%) | 3 (7.5%) | 7 (5.7%) |  |
| Abrasion | 8 (4.9%) | 2 (5.0%) | 6 (4.9%) |  |
| Amputation | 2 (1.2%) | 1 (2.5%) | 1 (0.8%) |  |
| Broken bone | 0 (0%) | 0 (0%) | 0 (0%) |  |
| Bruise | 0 (0%) | 0 (0%) | 0 (0%) |  |
| Concussion | 0 (0%) | 0 (0%) | 0 (0%) |  |
| Unknown | 19 | 10 | 9 |  |
| Mechanism of injury |  |  |  | 0.5 |
| Struck | 130 (72%) | 39 (80%) | 91 (69%) |  |
| Other | 26 (14%) | 7 (14%) | 19 (15%) |  |
| Fall | 11 (6.1%) | 2 (4.1%) | 9 (6.9%) |  |
| Burn | 6 (3.3%) | 1 (2.0%) | 5 (3.8%) |  |
| Caught in between | 7 (3.9%) | 0 (0%) | 7 (5.3%) |  |
| Unknown | 2 | 1 | 1 |  |
| ^1^n (%) | | | | |
| ^2^Fisher's exact test | | | | |

| Table 8: Injury characteristics among new occupational health patients <18 years by age, MSF clinics, Dhaka, 2014-2023   \| **Characteristic** \| **Overall**, N = 182^1^ \| **<14**, N = 50^1^ \| **14-17**, N = 132^1^ \| **p-value**^2^ \| \| --- \| --- \| --- \| --- \| --- \| \| Type of factory \|  \|  \|  \| 0.9 \| \| Metal \| 119 (65%) \| 35 (70%) \| 84 (64%) \|  \| \| Plastics \| 36 (20%) \| 8 (16%) \| 28 (21%) \|  \| \| Garment \| 13 (7.1%) \| 4 (8.0%) \| 9 (6.8%) \|  \| \| Embroidery \| 7 (3.8%) \| 1 (2.0%) \| 6 (4.5%) \|  \| \| Other \| 6 (3.3%) \| 2 (4.0%) \| 4 (3.0%) \|  \| \| Leather \| 1 (0.5%) \| 0 (0%) \| 1 (0.8%) \|  \| \| Rubber \| 0 (0%) \| 0 (0%) \| 0 (0%) \|  \| \| Tannery \| 0 (0%) \| 0 (0%) \| 0 (0%) \|  \| \| Type of injury \|  \|  \|  \| 0.3 \| \| Cut/laceration \| 100 (61%) \| 28 (70%) \| 72 (59%) \|  \| \| Other \| 29 (18%) \| 3 (7.5%) \| 26 (21%) \|  \| \| Crushing injury \| 14 (8.6%) \| 3 (7.5%) \| 11 (8.9%) \|  \| \| Burn \| 10 (6.1%) \| 3 (7.5%) \| 7 (5.7%) \|  \| \| Abrasion \| 8 (4.9%) \| 2 (5.0%) \| 6 (4.9%) \|  \| \| Amputation \| 2 (1.2%) \| 1 (2.5%) \| 1 (0.8%) \|  \| \| Broken bone \| 0 (0%) \| 0 (0%) \| 0 (0%) \|  \| \| Bruise \| 0 (0%) \| 0 (0%) \| 0 (0%) \|  \| \| Concussion \| 0 (0%) \| 0 (0%) \| 0 (0%) \|  \| \| Unknown \| 19 \| 10 \| 9 \|  \| \| Mechanism of injury \|  \|  \|  \| 0.5 \| \| Struck \| 130 (72%) \| 39 (80%) \| 91 (69%) \|  \| \| Other \| 26 (14%) \| 7 (14%) \| 19 (15%) \|  \| \| Fall \| 11 (6.1%) \| 2 (4.1%) \| 9 (6.9%) \|  \| \| Burn \| 6 (3.3%) \| 1 (2.0%) \| 5 (3.8%) \|  \| \| Caught in between \| 7 (3.9%) \| 0 (0%) \| 7 (5.3%) \|  \| \| Unknown \| 2 \| 1 \| 1 \|  \| \| ^1^n (%) \| \| \| \| \| \| ^2^Fisher's exact test \| \| \| \| \| |
| --- | --- | --- | --- | --- | --- | --- | --- | --- | --- | --- | --- | --- | --- | --- | --- | --- | --- | --- | --- | --- | --- | --- | --- | --- | --- | --- | --- | --- | --- | --- | --- | --- | --- | --- | --- | --- | --- | --- | --- | --- | --- | --- | --- | --- | --- | --- | --- | --- | --- | --- | --- | --- | --- | --- | --- | --- | --- | --- | --- | --- | --- | --- | --- | --- | --- | --- | --- | --- | --- | --- | --- | --- | --- | --- | --- | --- | --- | --- | --- | --- | --- | --- | --- | --- | --- | --- | --- | --- | --- | --- | --- | --- | --- | --- | --- | --- | --- | --- | --- | --- | --- | --- | --- | --- | --- | --- | --- | --- | --- | --- | --- | --- | --- | --- | --- | --- | --- | --- | --- | --- | --- | --- | --- | --- | --- | --- | --- | --- | --- | --- | --- | --- | --- | --- | --- | --- | --- | --- | --- | --- | --- | --- | --- | --- | --- | --- | --- | --- | --- | --- |
